# Primary Psychosis: Risk and Protective Factors and Early Detection of the Onset

**DOI:** 10.3390/diagnostics11112146

**Published:** 2021-11-19

**Authors:** Claudio Brasso, Benedetta Giordano, Cristina Badino, Silvio Bellino, Paola Bozzatello, Cristiana Montemagni, Paola Rocca

**Affiliations:** Department of Neuroscience “Rita Levi Montalcini”, University of Turin, Via Cherasco n. 15, 10126 Turin, Italy; claudio.brasso@unito.it (C.B.); benedetta.giordano@unito.it (B.G.); cristina.badino@unito.it (C.B.); silvio.bellino@unito.it (S.B.); paola.bozzatello@unito.it (P.B.); cristiana.montemagni@libero.it (C.M.)

**Keywords:** schizophrenia, prodromal stage, risk stratification, onset, early detection, prevention, incidence, duration of illness (DOI), duration of untreated psychosis (DUP), prognosis

## Abstract

Primary psychosis, which includes schizophrenia and other psychoses not caused by other psychic or physical conditions, has a strong impact worldwide in terms of disability, suffering and costs. Consequently, improvement of strategies to reduce the incidence and to improve the prognosis of this disorder is a current need. The purpose of this work is to review the current scientific literature on the main risk and protective factors of primary psychosis and to examine the main models of prevention, especially those related to the early detection of the onset. The conditions more strongly associated with primary psychosis are socio-demographic and economic factors such as male gender, birth in winter, ethnic minority, immigrant status, and difficult socio-economic conditions while the best-established preventive factors are elevated socio-economic status and an economic well-being. Risk and protective factors may be the targets for primordial, primary, and secondary preventive strategies. Acting on modifiable factors may reduce the incidence of the disorder or postpone its onset, while an early detection of the new cases enables a prompt treatment and a consequential better prognosis. According to this evidence, the study of the determinants of primary psychosis has a pivotal role in designing and promoting preventive policies aimed at reducing the burden of disability and suffering of the disorder.

## 1. Introduction

Primary psychosis is a heterogeneous group of mental disorders that includes various diagnoses characterized by positive symptoms (i.e., delusions and/or hallucinations), disorganized thinking and/or motor behavior, and negative symptoms (i.e., affective blunting, alogia, asociality, anhedonia, and avolition) [1]. The term primary indicates that these disorders are not secondary to the effects of a psychoactive drug (substance or medication) or to another pathological condition (e.g., neoplasia, autoimmune disease, depression, mania) [1]. Two main diagnostic systems are employed in psychiatry: the Diagnostic and Statistical Manual of mental disorders, 5th edition [2] and the International Classification of Diseases 11th Revision [3]. Both include diagnoses that meet the above-mentioned diagnostic criteria for primary psychosis. In the DSM-5 [2] these diagnoses are schizophrenia, schizophreniform disorder, brief psychotic disorder, schizoaffective disorder, delusional disorder, other specified schizophrenia spectrum and other psychotic disorder, and unspecified schizophrenia spectrum and other psychotic disorder, while in the ICD-11 [3] schizophrenia, acute and transient psychotic disorder, schizoaffective disorder, delusional disorder, and other primary psychotic disorder. The lifetime prevalence primary psychosis is around 1.94% [4] and schizophrenia, the most prevalent disorder of this group of diagnoses, is the 19th disease in terms of years lived with disability in both genders worldwide [5].

The onset of primary psychosis usually occurs in adolescence or early adulthood and men are more often diagnosed earlier than women: 18–25 years old men and 25–35 years old women [6]. The onset can be preceded by premorbid developmental alterations and can start with a prodromal phase characterized initially by non-specific symptoms and then by subthreshold positive, negative, affective, and behavioral symptoms. The prodromal phase can be absent or not identifiable. Then, the first expression of psychotic symptoms appears, followed by an increase in characteristic symptoms resulting in a definite diagnosis of primary psychosis [1]. According to the speed of appearance of psychotic symptoms, three modes of onset of primary psychosis have been identified: acute (psychotic symptoms appear from few hours to one month), gradual (from one to six months), and insidious (more than six months) [7].

A correct and early diagnosis is necessary in order to set up appropriate treatments promptly and thus to reduce the duration of untreated psychosis (DUP). This a key point in the treatment of primary psychosis, as a shorter DUP has been associated with improved prognosis in terms of less severe positive, negative, and global psychopathological symptoms, a higher chance of remission, and a higher overall functioning [8,9,10]. In addition, a longer duration of illness (DOI), defined as the time elapsed since the onset of prodromal symptoms, is associated with poorer treatment response, higher suicidal risk, and lower social functioning [11]. Therefore, acting in order to postpone the onset of the prodromal phase, thus shortening the DOI, could improve the prognosis of primary psychosis. Given the necessity to reduce the burden of disability related to primary psychosis, an implementation of preventive strategies in this field is needed. One possible way to achieve this is to focus on the study of the trajectories that leads to primary psychosis. Even though little is clearly known about the etiology and pathophysiology of the disorder, a wide field of the scientific literature has studied possible determinants of primary psychosis in terms of statistical association, namely risk and protective factors. The in-depth knowledge of these determinants can be employed to implement prevention programs and health promotion. Indeed, acting on modifiable risk and protective factors with prevention and health promotion policies might help to reduce the disability, economic, and emotional burden of primary psychosis. This could happen in two ways: on the one hand, it might reduce or delay the onset of the disorder, thus decreasing lifetime prevalence and the DOI; on the other hand, it enables to formulate risk algorithms to identify promptly new cases of primary psychosis, hence reducing the DUP.

In light of this, the purpose of this work is to review the main and most up-to-date scientific evidence on the risk and protective factors for the development of primary psychosis by evaluating them as possible targets or instruments for prevention and early detection of the onset.

Considering the objective of the present review, the main hypothesis underlying is that a deeper knowledge of risk and protective factors in this field may broaden the understanding of the mechanisms that lead to primary psychosis, allowing in this way to carry out more aware, targeted and effective preventive policies.

## 2. Methods 

We searched the Web of Knowledge database (incorporating Web of Science and MEDLINE) using the following search string: ((((TI = (“protective factors”)) AND TI = (psychosis)) OR (TI = (“protective factors”)) AND TI = (schizophrenia)) OR (TI = (“risk factors”)) AND TI = (schizophrenia)) OR (TI = (“risk factors”)) AND TI = (psychosis) OR ((((TI = (“prevention”)) AND TI = (psychosis)) OR (TI = (“prevention”)) AND TI = (schizophrenia)) OR (TI = (“detection”)) AND TI = (schizophrenia)) OR (TI = (“detection”)) AND TI = (psychosis). We selected papers published from 1980 and 16 August 2021. No proximity searches nor hyphen were employed. All kind of articles were included in the research, even if mainly second literature publications (reviews, systematic reviews, meta-analyses, umbrella reviews and meta-umbrella reviews). Original articles were included in two cases:

(i) they were very recent and were not cited in second literature publications;
(ii) They discussed topics never included into second literature works, mainly regarding protective factors or preventive programs that have a less wide literature.

**Figure 1:**
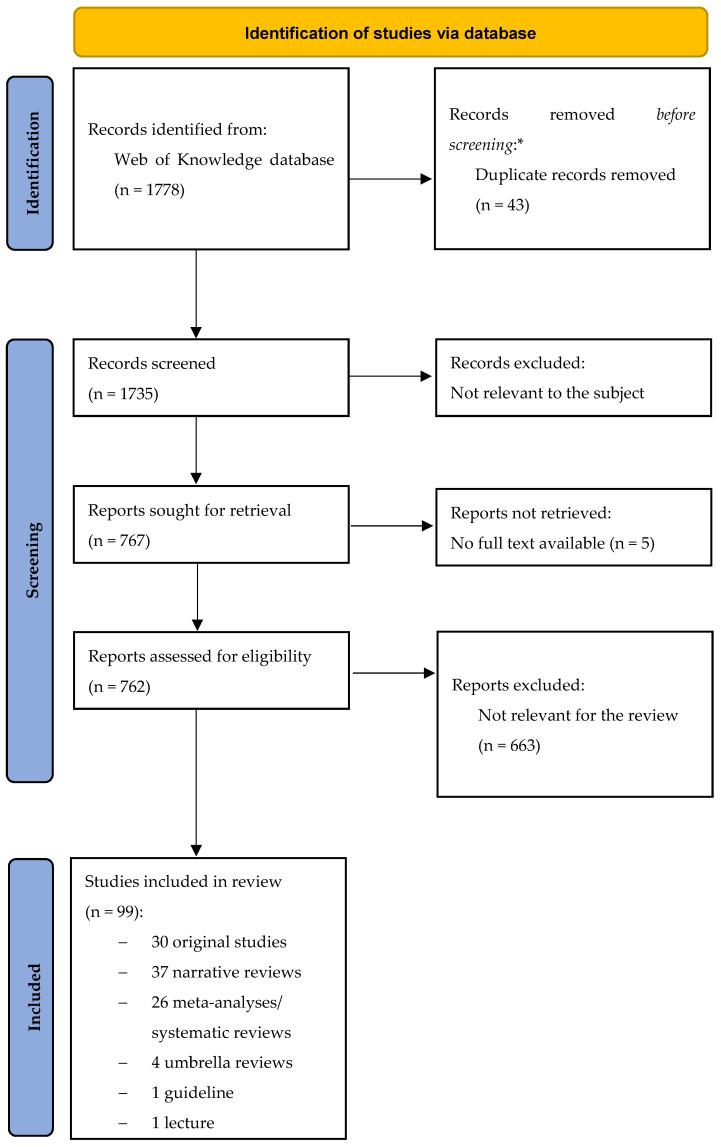
PRISMA flow-chart. * No automation tools were used. From: The PRISMA 2020 statement: an updated guideline for reporting systematic reviews [12].

We excluded original articles and reviews/meta-analyses/systematic reviews if already comprised is systematic syntheses like umbrella and meta-umbrella reviews, unpublished works (e.g., posters) and publications written in a language other than English and German for the second part. The selection process is shown in Figure 1.

We divided this narrative review into the following two parts: a description of risk and protective factors for primary psychosis and a discussion on the potential role of these determinants in prevention strategies, particularly in the early detection of the onset.

The choice to discuss both risk and protective factors and early detection of the onset of primary psychosis is motivated by the fact that risk and protective factors could allow an earlier identification of subjects with primary psychosis (secondary prevention) and that policies aimed at enhancing the disorder’s prognosis act at the level of risk and protective factors themselves (primordial and primary prevention). However, in scientific literature the two topics are often discussed separately, so we decided to structure results section in two separate parts, in order to improve the clarity of the text.

## 3. Results

### 3.1. Risk Factors for Primary Psychosis

According to the WHO definition, a risk factor is a determinant, namely any attribute, characteristic, or exposure that increases the likelihood of developing a disease, in this case primary psychosis, at an individual level. Risk factors can be divided into genetic and environmental factors. As for many other chronic diseases, genetic and environmental risk factors combine with each other increasing the likelihood of developing primary psychosis. In particular, gene-environment (GE) interplay can be classified into GE interaction (GxE) and GE correlation (rGE). GE will be used to indicate a general interplay between genetic and environmental factors, GxE to describe a random relationship, and rGE a non-random interaction where the presence of a specific genetic factor increases the likelihood of being exposed to a specific environmental determinant. rGE can be passive (e.g., the association between genetic factors transmitted by the parents to the offspring and an abusive home environment that can traumatize the offspring itself), evocative (e.g., genetic factors can lead to childhood anxiety disorders and consequently to social rejection trauma) and active (e.g., genetic factors can facilitate the use of cannabis) [13]. 

Thus far, it is not possible to address genetic risk factors in the offspring; however, some environmental risk factors are modifiable and offer the opportunity to act on them in terms of prevention. A determinant that can be modified by intervention, thereby reducing the probability of occurrence of disease or other outcomes (e.g., delay the onset) may be referred to as a modifiable risk factor, and logically must be a cause of the disease [14]. On the contrary, an attribute that that can be used as an indicator of increased risk of developing a disease but is not necessarily a causal factor is referred to as risk marker. In primary psychosis, risk markers are usually the result of different interacting genetic and environmental risk factors. Some examples are ultra-high-risk state, ethnicity, and immigration status [15].

In the next two sub-sections, we will present the main risk and protective factors for the development of primary psychosis. To improve expository clarity and facilitate the reading of this review, we will present the risk factors as part of two main categories: individual and environmental factors. This is an arbitrary distinction in which individual factors represent family or constitutive characteristics of the subject while environmental factors represent exposure to specific events or conditions. Genetic factors are part of the individual category. 

#### 3.1.1. Individual Risk Factors

##### Familiar and Parental Risk Factors

Family history is considered one of the strongest risk factors for primary psychosis [16], with a heritability (i.e., the degree to which a phenotype, in this case a disorder, is genetically determined, calculated by regression-correlation analyses among close relatives) of schizophrenia estimated around 79–81% and a similar proportion in other disorders of the spectrum [17,18]. In particular, for close biologic relatives of patients with schizophrenia [13,19,20] was demonstrated a 7.5-fold higher risk for the development of primary psychosis [21] and studies on monozygotic twins [13,19,20] showed a concordance rate of approximately 50% [20,22]. Other familiar risk factors for primary psychosis are a mother affected by primary psychosis, or a mood disorder, or any severe psychiatric condition or a father affected by any severe psychiatric condition [23]. 

Regarding parental factors, one of the most studied factors is parental age at the time of the birth of the children with a higher risk of developing primary psychosis in the offspring for maternal age <20 years or >30 years and a further risk for women with >35 years of age [23]. Similar associations were found for paternal age <20 years [15,23] and >35 years [23,24], with evidence of an increased risk for >45-years-old fathers [15]. Other parental factors associated with an increased o risk of developing primary psychosis are maternal pre-pregnancy obesity [15] or hypertension [23] and having more than three previous pregnancies [23], and low paternal socio-economic status [13,15].

##### Sociodemographic Risk Factors

Among all socio-demographic factors, it is well known that male individuals [15,20] with an age between 15 and 35 years [15] are at increased risk of developing psychosis. Moreover, being part of a disadvantage group and having a low socioeconomic status are two other risks factors for primary psychosis, as confirmed by different studies [13,15]. 

The season of birth is an additional important factor affecting the risk of psychosis [13]. In particular, winter season of birth [20,23] and winter/spring season of birth in Northern hemisphere [15,23,24] have been found to be strongly associated with an increased risk of developing primary psychosis. This risk appears to become stronger with the increase of latitude and the severity of winter [20]. However, the mechanism underneath is not totally understood, but it has been suggested that it relates to higher rates of prenatal infections and malnutrition [20].

Ethnicity represents one of the most implicated variables in the onset of primary psychosis. The concept of ethnicity includes two dimensions: having an immigrant status and belonging to a specific ethnic minority [15,24]. Both of these aspects may be better defined as risk markers as they may be the result of different interacting risk factors (e.g., racism, minority stress, isolation, low economic status etc.). In particular, the migrant status itself contributes to the pathogenesis of psychosis [13,15,20,24], mostly in second-generation immigrants [15,24]. For instance, being a Black-Caribbean ethnicity in England has the strongest statistical association [15,24], while other minorities like North African immigrants in Europe [15,24] and Black African and Asian ethnicity in England [15] showed weaker evidence. Different studies showed that migrants with black skin color might share a common risk exposure, possibly because of a genetic vulnerability background or for factors connected to ethnic disadvantage and lower socioeconomic status [25]. 

Finally, another important aspect in the development of primary psychosis is urbanicity: higher rates of psychotic disorders have been found in densely populated areas, suggesting that urban environments increase the risk of primary psychosis [13,15,20,24,26]. Urbanicity as well can be considered a risk marker as it acts through the interplay of multiple factors like deficit of green spaces, marginalization, and poor social network, as compared to rural areas [26]. Surprisingly, though, the effect of urbanicity does not seem to be widely mediated by pollution [27]. 

##### Personal Pre-Morbid Characteristics Associated with a Higher Risk of Developing Primary Psychosis

The following characteristics or conditions at birth have been associated with a higher risk of developing primary psychosis: premature birth [13,23]; use of incubator or resuscitation [15]; birth weight <2000 g [15], <2500 g [15,23], <2999 g [23], with an increased risk in terms of higher odd ratio (OR) for lower weights [13]; birth length less than 49 cm [23]; being small for gestational age [23]; and low or high levels of neonatal vitamin D [15]. 

Moreover, several secondary literature studies (e.g., umbrella reviews, meta-analyses) provided sufficient evidence about atypical developmental characteristics and a higher risk of developing primary psychosis. In particular, strong evidence was reported about minor physical defects [15,24]. Focusing on neurodevelopment, extra-cranial size, non-right handedness [15,24] and early life alterations in motor, cognitive, and language evolution seem to be associated with a higher risk of developing schizophrenia [28]. In particular, delay in walking unsupported [15,24], and pre-onset motor function alterations [15], showed some evidence regarding their role in increasing the risk of primary psychosis, however larger studies are required to confirm these associations. Focusing on intelligence, it has been identified a negative linear relationship between premorbid IQ deficit and the risk of developing schizophrenia [15,24,28], as for every 1-point decrease in premorbid IQ matches a 3.7% increase in risk of developing primary psychosis. In addition, greater IQ deficits are associated with an earlier illness onset [28]. 

As regards personality, many personality characteristics have been found to be implicated in the risk of developing primary psychosis, even though the majority of them showed a weak statistical association [15,16,24]. In particular, novelty seeking, harm avoidance, self-transcendence [15,24] and neuroticism [15,16] seem to increase the risk of developing primary psychosis, even if researchers agree that the most implied personality aspect is trait anhedonia [15,24].

##### Comorbidities Associated with a Higher Risk of Developing Primary Psychosis

Two groups of disorders appear to be more related to the onset of primary psychosis: autoimmune illnesses and psychiatric conditions. 

Focusing on the first group, there is an amount of evidence supporting the link between immunological processes and psychosis. Elevated levels of inflammatory markers, especially in childhood and adolescence, might increase the risk of primary psychosis. In addition, both primary psychosis and autoimmune diseases often show a relapse-remitting course of illness where recurrences are characterized by increments of peripheral inflammatory markers. Moreover, a family history of autoimmune disease seems to increase the risk of schizophrenia and associations with psychotic disorders have been found for several autoimmune diseases [29]. This link might be explained by an aberrant inflammatory response due to an immunogenetic predisposition that facilitate both autoimmune diseases and primary psychosis (e.g., common MCH genes) [29,30].

The vast majority of autoimmune diseases (e.g., multiple sclerosis [29], Systemic Lupus Erythematosus [29,31], autoimmune thyroid disorders like Grave’s disease [29,30,31], autoimmune encephalitis N-methyl-D-aspartate receptor–(NMDAR) antibody mediated [29,31], autoimmune hepatitis, Crohn’s disease, diabetes mellitus type 1, psoriasis and Guillain-Barre syndrome) can cause neuropsychiatric symptoms, including psychotic ones, with a direct pathophysiological mechanism. In these cases, psychosis can be considered secondary to autoimmune diseases themselves, which consequently cannot represent an actual risk factor for primary psychosis. On the other hand, some studies found that few autoimmune diseases could actually arise the risk of primary psychosis without a clear direct action on the CNS. Celiac disease seems to be associated with the risk of schizophrenia, since populations with smaller consumption of wheat seem to have lower incidence rates of schizophrenia, and gluten-free diet had positive effects on psychotic symptoms [29,30]. In addition, pernicious anemia and pemphigoid reached significant evidence regarding their role in increasing the risk of developing primary psychosis [30]. 

Regarding psychiatric comorbidities, a recent study found that premorbid eating disorders are common in people that will develop primary psychosis. Compared with patients without a history of eating disorders, these patients with primary psychosis present specific phenotypic features, that is female sex, higher IQ and more severe psychotic symptoms [32].

Other known psychiatric conditions associated with primary psychosis are Obsessive-compulsive disorder (OCD) and obsessive-compulsive symptoms (OCS). In particular, there is some evidence implying that a diagnosis of OCD may be associated with elevated risk for future development of primary psychosis and that OCD and OCS influence the course of the psychotic disorder [33].

Finally, schizotypal personality disorder seems to lie on a continuum with schizophrenia spectrum disorders and to represent a plausible factor somehow linked to primary psychosis [34,35,36]. The disorders seem to have a significant overlap in terms of etiology, as regards genetic, biological, and psychosocial factors [37].

##### Genetic Factors Associated with a Higher Risk of Developing Primary Psychosis 

Currently, primary psychosis, in particular schizophrenia, is considered polygenic and multifactorial disorders. People with primary psychosis often present multiple genetic polymorphisms quite frequent in the general population, each of which contributes a small effect to disease susceptibility. About this topic, in the last years, research focused on the identification of common genetic variants that play a role in the development of primary psychosis. However, despite genetics accounts for about 80% of the liability, no gene seems to be sufficient or necessary for the development of the disease [20].

Recently, due to the complex genetic architecture of primary psychosis and to the evolution of biotechnology, the focus has shifted from single genes studies to genome-wide scale (Genome Wide Association Studies—GWAS). For example, the study conducted by the Schizophrenia Working Group identified more than 100 independent loci associated with schizophrenia [38], suggesting that these variants contribute to the genetic susceptibility for primary psychosis [13]. Simultaneously, the detection of chromosomal rearrangements pointed out the implication of copy number variants—CNVs, small insertions and deletions (indels), as for the 22q11.2 microdeletion syndrome [31,39,40,41]. Compared to single CNVs and indels, a smaller contribution to the risk of developing primary psychosis was found for each single nucleotide variation—SNV [17,19,39]. CNVs and indels are unique and rare in the general population. Instead, common in individuals with neurodevelopment disorders, including primary psychosis [17,40]. In fact, approximately 2.5% of schizophrenia cases have a known CNV [40], suggesting that these specific genetic variants might take part in the pathogenesis of primary psychosis. 

The possible role of the product of gene loci associated with a higher risk of developing primary psychosis are described in Table 1 [17,18,22,42,43]. Genes were grouped according to the main function of their product. 

It should also be emphasized that some genetic variants are implicated in increasing the risk of psychosis not only for themselves but also mainly through the association with certain environmental risk factors. For example, specific genetic variants of the Catechol-O-Methyltransferase (COMT) increase the risk of developing primary psychosis mainly in subjects exposed to childhood trauma or stress. Other similar GE interplay are those of FK Binding Protein Prolyl Isomerase 5 (FKBP5) and childhood trauma, AKT-8 retrovirus Serine/Threonine Kinase 1 (AKT1) and cannabis use, and Catenin Alpha 3 (CTNNA3) and cytomegalovirus infection in utero [13].

At last, some evidence supported the role in the pathogenesis of primary psychosis of specific gene variants coding for molecules related to the functioning of the immune system. In particular, alterations in neuroinflammatory pathway like microglial activation are associated with primary psychosis [17]. On a molecular level, these processes are mainly supported by imbalance in inflammatory cytokines (altered signaling of interleukin 1 beta-IL-1B—and increased levels of IL-6) and alterations in the nuclear factor kappa-light-chain-enhancer of activated B cells (NF-kB) signaling [17]. Moreover, focusing on adaptive immunity, the most studied loci in primary psychosis, especially in schizophrenia, are HLA- A, B, and C of the antigen-presenting Major Histocompatibility Complex (MHC), Class I and HLA-DRB1 and DQB1 of the MHC, Class II. Many different variants of these loci were associated with the diagnosis of a primary psychosis [17,19].

#### 3.1.2. Environmental Risk Factors

##### Prenatal and Perinatal Risk Factors

Prenatal and perinatal environment, and consequently insults, is largely implicated in the etiopathogenesis of primary psychosis, even though its specific contribution remains unclear [23]. Recent meta-analyses and umbrella reviews have revealed that several pregnancy-related and perinatal risk factors are significantly associated with psychosis [13,15,23,24,48], suggesting the possible role of suboptimal number of antenatal care visits on mother’s mental health [23]. Obstetric complications represent some of the most studied and most consistently implicated environmental risk factors. Among them, worth mentioning are diabetes or obesity during pregnancy [15]; emergency caesarean section [15]; threatened premature delivery [15]; uterine atony [15]; unspecified obstetric complications [13,15,23,24]; antepartum hemorrhage [15,23]; hypoxia [23]; preterm ruptured membranes [23]; polyhydramnios [23]; HSV type 2, Toxoplasma gondii or other unspecified maternal infections [13,23,48], especially during the second trimester [48]; maternal anemia [13]; maternal stress not otherwise specified [13,23]; maternal nutritional deficits [13,23]; asphyxia during delivery [23].

In addition, Ursini et al. [49] pointed out that the interactions between some set of genes that participate in the cellular stress response and are selectively expressed in the placenta and the exposure of specific intra-uterine complications (e.g., prenatal infection) may increase the risk of developing primary psychosis. The Authors suggest that this adverse prenatal GE interplay could negatively influence placental transcriptome and thus placental functioning and fetal development. This vulnerability is higher for male fetuses. 

In terms of pre and perinatal risk factors, it has been suggested that the activation of the immune system, through oxidative stress and neuroinflammation, could mediate their effects on psychotic risk, supporting the abnormal neurodevelopment. In fact, subjects exposed to high inflammation in utero are at an increased risk of developing schizophrenia in the future [13]. In particular, N-methyl-D-aspartate receptors (NMDAR) located in a subpopulation of cortical parvalbumin-containing interneurons (PVIs) might be more sensitive to neuroinflammation during the perinatal and pubertal periods, resulting in a hypofunction and in a synaptic alteration [50]. 

##### Childhood-Related Environmental Risk Factors

In the last decades, early traumas have reached a particular research interest in mental health. In particular, trauma models of mental disorders have highlighted the effect of different kinds of stress during the early stages of life as key factors in the future development of psychiatric conditions [51].

Focusing on the risk of developing primary psychosis, strong evidence has been found about childhood adversities and traumas in general [24,51]. Childhood adversities include child maltreatment (sexual, physical and emotional/psychological abuse, neglect) [13,15,52,53,54], peer victimization (in particular bullying) [13,52,53], both active and passive [55], parental death [52,53], and parental separation [55].

Only parental death appears to be independently associated with augmented primary psychosis risk [52]. The other factors seem to interact with each other and with other variables such as age, frequency and duration of the exposure to the traumas in determining the increment of the risk. In this view, increasing attention has been paid to dose-response effects of trauma. With regard to these aspects, it should be stressed that these types of risk factors tend to add to each other as being exposed to one type of traumatizing event actually increases the risk of being expose to another [53].

Focusing on the GE interplay of early life traumas, different studies reported an rGE correlation showing that the offspring of a parent affected by psychosis is more prone to be exposed to dysfunctional elements of the family environment, as in the case of parental communication deviance [16]. Moreover, the effect of childhood trauma in increasing the risk or psychosis has been found to be partially independent from a previous genetic susceptibility [56]. One possible mechanism underlying this partial independence of the environmental factors from the genetic ones is the epigenetic impact of childhood traumas on gene expression profile, in particular through altered global DNA mutilation [57]. According to this explanation, there would be no need of an alteration of the DNA sequence for a variation of gene expression such that a phenotype of primary psychosis emerges, as epigenetic effects of traumas would be sufficient by themselves. 

Another risk factor to be considered is the exposure to infections during childhood, suggesting that the period of vulnerability to infections is not confined to the prenatal one. Viral infections involving central nervous system (CNS) are associated with a higher risk of subsequent psychosis [28,31,58]. Moreover subjects with multiple hospitalizations for infections have a higher risk of developing psychosis [58]. Two main hypotheses have been proposed to explain this relationship, that are a direct and an indirect brain damage. The first is directly caused by the pathogen, while the second one is mediated by a dysfunctional activation of the immune system in the CNS after the pathogen stimulus [58]. Following this latter hypothesis, besides CNS infections, other infections (e.g., Toxoplasma gondii IgG+, Toxocara spp, Chlamydia psittaci, Human endogenous retrovirus type W, Chlamydia pneumoniae, Borna disease virus, Human Herpes Virus type 2), occurring after birth and before the onset of psychotic symptoms, represent risk factors for psychosis [15,24]. 

##### Adolescence-Related Environmental Risk Factors 

Environmental risk factors associated with primary psychosis that occur later in development, usually during adolescence, are mostly substance use-related [13,20]. 

In particular, cannabis use has been strongly associated with primary psychosis [20,24,56,59,60]. Cannabis users have an increased risk of developing schizophrenia [13], proportionally to the cumulative amount consumed [13,59,61]. In addition, cannabis abuse is associated with an earlier onset of the disorder [56]. Another factor influencing the risk related to cannabis abuse is the age of onset of cannabis consumption, as early consumption, particularly before 15 years of age, results in a greater likelihood of developing primary psychosis during adulthood [61]. This association between cannabis use and risk of developing primary psychosis can be another example of a GE interplay; in fact, the presence of a specific polymorphism in the COMT gene, which reduces the activity of the enzyme, increases the risk of developing primary psychosis in cannabis users [61].

Other substances associated with the development of psychosis are psychostimulants. The abuse of these drugs (above all cocaine and amphetamines) is usually associated with acute psychosis, with a four-time increased risk; furthermore, subjects with a family history of psychiatric illness that abuse psychostimulants appear to be more susceptible to persistent psychotic symptoms. Moreover, this connection between psychostimulants and risk of psychosis can be also applied to children with a family history of mental disorders taking these drugs to treat attention-deficit/hyperactivity disorder (ADHD) [13].

Tobacco as well could take part in psychosis risk [13,15,24,62] with a 2-fold increase in risk of schizophrenia or other schizophrenia spectrum disorders and a dose-response mechanism [63]. The mechanism underlying this association is not clear, with some hypotheses in favor of an rGE correlation where a genetic arrangement that favors the development of primary psychosis also favors tobacco smoking for attenuating a specific distress that manifests in the prodromal phase of the disorder. In any case, a clear causal link between cigarette smoking and the development of primary psychosis has not yet been established.

Finally, among substance abuse, alcohol is well known to be associated with neuropsychiatric consequences, including delirium tremens, alcohol-related brain damage, Korsakoff’s syndrome and alcoholic hallucinosis [64]; meanwhile individuals with psychotic disorders, in particular schizohrenia or schizoaffective disorder, have a three times increased risk of severe alcohol use [65,66], link which is partially explained by a shared genetic liability [67]. 

In contrast to other substances listed before which have evidence in increasing risk of primary psychosis, in literature there are no findings about a similar role of alcohol or its role in transition to psychosis in clinical high-risk patients [68], even if secondary psychotic symptoms are relatively frequent.

In addition, some studies highlighted a possible link between maternal alcohol use during pregnancy and different psychopathological outcomes in the offspring, including primary psychosis [69,70], but no statistically significative association has been found so far.

##### Continuous Risk Factors and Factors That Can Play a Role at Any Time in Life

As regards environmental pollutants, there is evidence that exposure to benzene [15], xenobiotic heavy metals (e.g., lead and cadmium), nitrogen and sulfur oxides, organic solvents, and other components of may increase the risk of developing primary psychosis [13,71]. The neurobiological mechanism underlying this connection is not clearly known.

Traumatic brain injury can play a role in facilitating the onset of primary psychosis at any time in life [13,15,72]. In this case, the two main mechanisms proposed to explain this connection are that the permanent damage caused by the trauma alters the CNS, favoring the onset of the disorder and that subjects with schizophrenia prodromes may be more exposed and sensitive to accidents [72].

Finally, considering that last years’ SARS-CoV-2 pandemic, some studies have investigated the relationship between COVID-19 respiratory infection and the onset of psychosis. The results have been summarized in a meta-analysis by Watson et al. [73], which showed that infected patients can present a range of neuropsychiatric symptoms but did not find any significant strong association between the SARS-CoV-2 infection and primary psychosis.

### 3.2. Protective Factors for Primary Psychosis

Protective factors have been defined as “those factors that modify, ameliorate or alter a person’s response to some environmental hazard that predisposes to a maladaptive outcome” [74]. Regarding their role, they do not necessarily promote normal evolution in the absence of risk factors, but they may make a remarkable difference in the influence of risk factors [75]. Notably, a positive environment might play a role in protecting genetically susceptible individuals, especially during the vulnerable adolescence period [16].

Compared to risk factors, relatively few studies concerning protective factors for primary psychosis have been carried out during the years. 

As for the previous discussion about risk factors, we will present protective factors as part of two main categories: individual and environmental factors.

#### 3.2.1. Individual Protective Factors

##### Parental, Socio-Demographic, and Personal Protective Factors

Regarding maternal protective factors, worth mentioning are maternal age between 20–29 years and maternal nulliparity [23]. 

Some socio-demographic factors are implied in protecting from the risk of developing psychosis, like an age >35 years and an elevated socio-economic status [15].

Among personal physical characteristics, also, in this case, birthweight plays a part as higher birthweight, in particular if >3500 g [23], represent a protective factor against psychosis.

Furthermore, higher olfactory identification abilities seem to be protective against psychosis [15,24]. This connection is motivated by the fact that in patients with schizophrenia have been observed important olfactory deficits in different olfactory tasks, in particular, odor memory and identification. This is a result of a dysfunction in the peripheral and central olfactory system, i.e., the primary olfactory cortex. This alteration may also involve other brain regions associated with the pathophysiology of the disorder, like the temporal lobe [76]. These olfactory deficits have not exceeded the statistical threshold to be considered valid risk factors for the development of primary psychosis [15,24]. On the contrary, however, more developed olfactory abilities seem to protect from the disorder [15,24]. 

As mentioned above, premorbid IQ plays a role in the pathogenesis of primary psychosis. It has been observed that individuals with low or average IQ were at significantly increased risk of developing schizophrenia than those with higher IQ, suggesting the active and protective role of cognitive reserve in primary psychosis [24,28], even in case of 12-years old subjects with a positive family history for psychosis [77].

The role of personality in psychopathology has long been explored and some personality traits, such as extraversion, openness, agreeableness, and conscientiousness have been found to be negatively associated with psychosis [15,16].

Even though resilience is a heterogeneous concept difficult to assess, it is clear that it is a quality that contributes to positive adaptation to significant adversities and demanding ambient [16,78]. Resilience in some high-risk individuals appeared to be protective against developing schizophrenia, but the reason remains unknown and needs further investigations [78].

##### Autoimmune Comorbidities Associated with a Reduced Risk of Developing Primary Psychosis

Regarding general comorbidities, among autoimmune disorders, recent research has found significant negative associations between rheumatoid arthritis [29,30,79] and ankylosing spondylitis [29,30] and primary psychosis. 

Due to the late onset of rheumatoid arthritis, is has been suggested that this relationship can be partly explained by an under-diagnosis, caused by a limited life expectancy and worse health care in individuals with psychosis [30]. However, a specific MHC class II DRB1 gene variant, namely the HLA-DRB1*04, has been associated with both an increased risk of rheumatoid arthritis and a reduced risk of developing schizophrenia [17,18,30], suggesting that a common immunogenetic background may promote the autoimmune disorder and protects from the mental one. 

As concerns ankylosing spondylitis, the age of onset is far earlier, so that the under-diagnosis explanation due to the age of onset of the autoimmune disease in relation to that of primary psychosis cannot be given. Moreover, apparently, the two disorders do not seem to share a clear common immunogenetic profile; therefore, further investigations are needed [30]. 

#### 3.2.2. Environmental Protective Factors

##### Prenatal Protective Factors

As nutritional deprivation during pregnancy has been found to increase risk of psychosis, as well as an adequate prenatal nutrition, are essential to satisfying the increased nutrient demand and guarantee a normal fetal development [80]. In this context, prenatal dietary supplementation with vitamin D, iron and folates [80], or phosphatidyl-choline [81] seem to help preventing psychosis onset. 

Furthermore, a recent study pointed out that individuals exposed to elevated maternal levels of anti-inflammatory cytokines whose production was stimulated by T helper 2 lymphocytes in utero, were significantly less prone to evolve in psychosis in adulthood [82].

##### Childhood and Adolescence-Related Environmental Protective Factors 

The role of family environment is implicated also in protecting against the onset of psychosis. Strong evidence has been reported about its protective role. In particular a family with four children or less, with two or more years difference between them, a good parental system, clear boundaries within the family and healthy relationships with relatives appear to be protective against primary psychosis [16]. A more positive atmosphere at home [83,84], the perception of an elevated social support among 18-years-old people with mothers with psychosis-spectrum disorder [77] and a greater neighborhood social cohesion among children of mothers with a psychosis-spectrum disorder [77] were found to be protective against the psychosis onset in the offspring in that defined group of patients.

Living in a stimulating family environment and in a cohesive community were found to be protective factors against primary psychosis [16], even among children exposed to poly-victimizations, while social support and living in a cohesive community are protective for poly-victimized adolescents [83].

Finally, among studies on adopted individuals, healthy patterns of baby growth [85] and a healthy and great communication between adopting parents and children [86] reduce the risk of primary psychosis in subjects at high genetic risk for schizophrenia. 

A recent meta-analysis of Brokmeier et al. [87] analyzed the role of physical activity as a risk or protective factor in incidence of psychosis. A more frequent physical activity is associated with a 27% decreased risk of psychosis, though the sample size was limited, so this result should be confirmed by larger studies.

A summary of risk and protective factors and markers, divided according to the phase of occurrence and their putative role in preventive strategies is shown in Table 2.

### 3.3. Early Detection of the Onset

The onset of psychosis can be defined as an acute phase crisis characterized by florid and sustained psychotic symptoms (delusions and/or hallucinations), lasting at least four weeks [91]. Over the past two decades, increasing efforts have been made to detect early the onset of psychosis to minimize the DUP and improve clinical outcomes. This leads to preventive strategies with the aim to postpone and promptly detect the onset. In order to achieve this goal, it is necessary to identify populations at risk and stratify the risk through apposite tools in order to carry out targeted interventions. In accordance with this, we will present the main populations at risk, the tools used for the stratification of the risk itself, and possible models of preventive interventions.

#### 3.3.1. Help Seekers and Populations at Risk of Developing Primary Psychosis

In the context of the general population, where the lifetime risk of developing schizophrenia is about 1%, it is possible to find individuals seeking help at early detection services. The so-called “help-seekers” usually have been exposed to multiple risk factors and have an increased risk of developing primary psychosis. However, only a 20–30% of these subjects meets the criteria for the Clinical High Risk for Psychosis (CHR-P) state, defined by the concomitance of certain risk factors, a functional impairment, and possible associated attenuated psychotic symptoms [92]. In any case, a follow-up for help-seekers is required as the CHR-P subpopulation has a 20% risk of developing psychosis in 2 years (first episode of psychosis or persistent attenuated psychotic syndrome) while another 60–70% of the help-seekers will face another psychiatric disorder (e.g., bipolar disorders, major depressive disorder, substance use disorders, personality disorders etc. [92,93]

Moreover, among CHR-P subjects has been identified a further subgroup called Ultra High Risk (UHR), whose criteria currently require to have had one of this conditions: attenuated psychotic symptoms during the past year (APS); brief, limited or intermittent psychotic symptoms (BLIPS), not lasted longer than a week and spontaneously remitted; a first degree relative affected by a psychotic disorder or a schizotypal personality disorder, associated with a significant functional impairment during the previous year [94]. The transition rate to full-blown psychosis of these subjects was estimated at around 30% in two years [95]. 

#### 3.3.2. Screening Tools for Stratification of the Risk of Developing Primary Psychosis

Several kinds of instruments have been proposed to enhance prediction of psychosis onset in the CHR-P population. In particular, different clinical screening questionnaire have been suggested such as the Prodromal Questionnaire [96], which focuses on the prodromal phase, and the Clinical High At Risk Mental State (CHARMS) developed by McGorry et al. [93]. The risk calculator developed by Cannon et al. [97] and the trans-diagnostic risk calculator by Fusar-Poli et al. [98], though, are screening based on risk identification approaches. Two main instruments for clinical translation of neuroimaging (PSYSCAN) and prognostic (PRONIA) information were also proposed [93]. In addition, probabilistic multimodal models combining different domains, that are patient history, clinical assessments, and biomarkers (e.g., neuroimaging, cognitive, and molecular markers), were also introduced to assess the risk of transition to overt primary psychosis [99,100]. The most known example is the model suggested by Clark and colleagues [99].

In addition, for the same purpose of risk stratification, in the last few years efforts have been made to take advantage of new technologies in order to improve the detection of new cases and the access to mental health (e.g., use of virtual reality, forum, online platforms, and mobile app specifically designed to identify risk factors and symptoms) [101,102]. These types of interventions, even promising, require more studies to be validated for the clinical practice. 

#### 3.3.3. Models and Levels of Preventive Interventions and their Limitation

Universal prevention at the level of general population promotes some pharmacological and psychosocial interventions acting on some well-known modifiable risk factors: perinatal administration of phosphatidylcholine and N-acetylcysteine to support brain development and anti-inflammatory neuroprotection; lifetime supplementation with omega-3 fatty acid, vitamins, sulforaphane and prebiotics to reduce neuroinflammation and enhance good mental health; school-based behavioral interventions to lower the risk of bullying and substance abuse; physical exercise to support cognitive functioning through improvement of neuronal plasticity [90].

Another level of prevention targets specific groups of individuals, whose risk of developing a mental disorder is significantly higher than the rest of the population, presenting an accumulation of risk factors. In these cases, school-based mental health assistance, assertiveness training, and stress and anxiety management have the greatest chance to prevent maladaptive behavior and symptomatic manifestations [90]. 

Anyway, nowadays the most employed preventive approach [94,103], focuses on subjects during prodromal stage, i.e., individual with CHR-P with the purpose to alter the trajectory of mental disorders, delaying the onset of first episode of psychosis, increasing the chance of better long-term outcomes, reducing unnecessary suffering caused by the delay of the intervention, and bettering engagement with services [103]. Therefore, enormous progress has been made in this field, and specific services have been implemented worldwide (Early intervention for psychosis services—EIS), targeted at young CHR/UHR people and their families [101,104].

Once identified CHR-P individuals, it is firstly indicated a primary prevention by the use of needs-based and psychological interventions (cognitive behavioral therapy—CBT or integrated psychosocial interventions, such as psychoeducation, social skills training, metacognitive training, cognitive remediation, and family therapy), that need to be characterized in the light of personal characteristics, risk profile (severity of attenuated positive and negative symptoms and level of functioning), preferences of the single subject [105]. Psychosocial interventions may be effective in decreasing vulnerability to psychosis; in fact, they increase coping mechanisms and lower vulnerability to stressors, improving self-confidence, self-esteem, cognitive abilities, social skills, social network, and support [106].

Among all interventions in reducing risk for transition in FEP at 12 and 18 months, Cognitive Behavioral therapy (CBT) showed the strongest level of evidence, being effective in restoring thoughts and changing behaviors that maintain symptomatology and distress, while other types of interventions, even promising, require more clinical trials [107]. Additionally, it is important to treat potential other comorbid psychiatric disorders following actual guidelines and aim to improve recovery and quality of life [105]. 

Although antipsychotic medications represent the indicated treatment for first episode psychosis (FEP), they are currently suggested in the prodromal phase of the illness only for specific complex cases (i.e., attenuated psychotic symptoms with an impact on global functioning and quality of life) and only for some low-dose atypical antipsychotics (mostly amisulpride), due to several potential side effects and to the lack of sound evidence of their effectiveness in preventing psychosis [106]. 

Antidepressant treatment is not indicated in FEP, even if there is some evidence that it might be successful in delaying the onset of psychosis, by treating depressive prodromal symptoms, as well as the use of anxiolytics may postpone psychosis [106]. 

Finally, there is some suggestion that CHR-P subjects may benefit from taking omega-3 polyunsaturated fatty acids (PUFAs), reducing the risk of transition to psychosis [106].

In addition to the mentioned established treatments historically used in CHR-P states, new recent advancements are under development and validation. Examples are exercise, smartphone applications, virtual reality, brain stimulation, social media, biofeedback, oxytocin-based interventions, mindfulness-based interventions, sleep hygiene, intervention enhancing protective factors, intervention eliminating barriers to health care, intervention reducing stigma, and mental health prevention in urban planning [102]. Nevertheless, studies regarding these recent research developments are still ongoing and it is necessary to wait for the results to have sufficient evidence and establish whether they could represent valid new treatment instruments. 

Despite the fact that recent studies have focused mainly on interventions during clinical high risk and prodromal stages [105,106], prospects of primary intervention are often limited by costs, practicability [108] and actual usefulness, due to the limited detection rate of the total onset (approximately 5%) [90]. 

#### 3.3.4. Integrated Model of Prevention and Early Detection of the Onset in Primary Psychosis

Greater hopes rest on preventive early interventions on the first episode of psychosis, aiming to postpone psychosis onset, reduce the duration of DUP, improve treatment response, well-being, functioning and social skills, and prevent disease progression [92]. Nevertheless, currently there is a lack of screening tools for the early identification of the onset of psychosis. The actual used psychopathological early recognition instruments (e.g., BSABS—Bonn Scale for the Assessment of Basic Symptoms [109], CAARMS—Comprehensive Assessment of At-Risk Mental States [110], SIPS—Structured Interview for Prodromal Syndromes [111]) mainly focused on assessing clinical symptoms. A particularly interesting instrument is the Early Recognition Inventory for the retrospective assessment of the Onset (ERIraos) Checklist, developed by Hafner and collaborators [112,113,114]. This is a two-step early recognition inventory, practical and sufficiently valid instrument to be used at a non-specialist primary care level, with good psychometric properties, meant for the early detection of UHR subjects and of patients with untreated illness, to assess the likelihood of psychosis onset. It is divided into two parts:

(i) a first part, the ERIraos-Check List, which is used for screening purposes, contains questions on generic and psychotic psychiatric symptoms, on global functioning, with importance given to self-perception of functioning, and on the evaluation of some well-known risk factors for primary psychosis such as obstetric complications, neurodevelopment delays or alterations, family history, and substance abuse;
(ii) a second part, the ERIraos-Symptom List, which is administered to people evaluated at least at modest risk of transition to psychosis in the ERIraos-Check List. This second-level evaluation is carried out in specialized centers and aims to carefully detect imminent transition [115]. Thereafter, the ERIraos has been successfully employed as a tool for the early recognition of psychosis risk in several further German studies [116,117,118] and subsequently translated and validated into different languages and populations [115,119]. This instrument proved to be suitable for routine use as a scale for the identification of the at-risk subjects.

Past strategies have been mostly based on finding programs and instruments designed to reduce the DUP [120,121,122]. For this purpose, a better collaboration between primary care services and specialist mental health services, which follow “consultation-liaison” and “collaborative care” models are needed to bring diagnostic tools in the context of primary care and create an integrated model of detection and prevention of primary psychosis [90].

An example of this strategy is the one carried out by Hafner et al. [108]. They suggested focusing on the estimated 70% of individuals with a DUP longer than one year, promoting in Germany a specific multi-step approach. First, at primary care centers they used the ERIraos Check List to identify subjects at risk of developing primary psychosis. These at-risk subjects were subsequently referred to an early-intervention center for a detailed risk assessment using the ERIraos Symptom List. This second assessment provided two risk profiles:

(i) subjects at early (pre-psychotic) prodromal stage and
(ii) subjects at late (early psychotic) prodromal stage.

For the treatment part of the project, subjects at early prodromal stage were randomized to cognitive behavioral psychotherapy (CBT) or usual clinical management, while those at late prodromal stage were randomized to antipsychotic treatment with amisulpride or usual clinical management. The work of Hafner and colleagues [108] represented an important pilot integrated approach, which evidenced promising even though limited results [123], but most of all prepared the ground for future similar integrated programs.

## 4. Discussion

As highlighted in the previous sections of this work, the risk and protective factors for the development of primary psychosis can be divided into modifiable and non-modifiable factors and risk factors proper and risk markers. Modifiable risk and protective factors allow to act on the incidence and prevalence of the disorder, those that cannot be modified and risk markers are useful for the stratification of the risk of developing primary psychosis and therefore for the design of screening tools. 

By acting on the modifiable factors that can actually influence the risk of developing primary psychosis, the incidence of the disorder can be reduced. These preventive interventions aimed at reducing incidence belong to primordial and primary prevention [14]. Similarly, acting on modifiable risk and preventive factors at a stage closer to the onset of primary psychosis the onset itself may be delayed, thus resulting in a reduction of the DOI. Through risk stratification and early identification of the onset, it will be possible to carry out a prompt and adequate treatment, thus reducing the DUP. This type of preventive interventions that can improve the prognosis of the disorder through the reduction of DOI and DUP belong to secondary prevention strategies [14]. 

**Figure 2:**
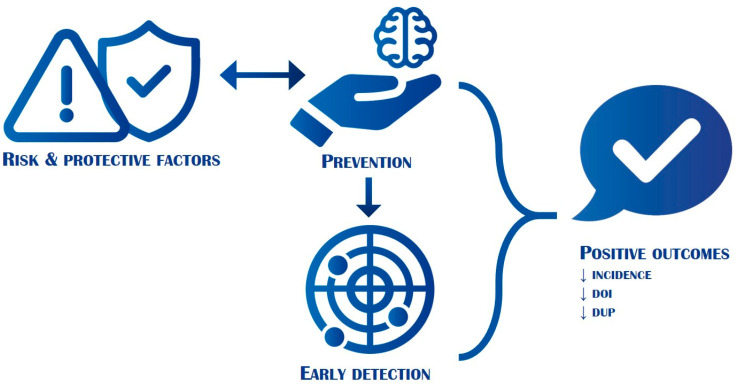
Interplay between risk and protective factors, prevention, early detection of the onset, and outcomes. DOI: duration of illness; DUP: duration of untreated psychosis. The figure shows how acting on risk and protective factors through prevention (horizontal arrow) and through an early detection of the onset (vertical arrow), is it possible to reduce the incidence, the DOI and the DUP of primary psychosis. This allows ameliorating the prognosis and lowering the global burden of disability of the disease (not shown).

All these preventive interventions have positive effects in terms of reduction of the burden of suffering and disability of the disorder and may improve the quality of life of patients with primary psychosis and their caregivers (Figure 2).

This is possible by implementing preventive and health promotion actions that reduce risk factors or promote protective ones. Such interventions may be broad-based and relevant for the general population, or more targeted and useful for specific subgroups of the population that may benefit from them.

In the first case, i.e., broad-based interventions, fall primordial preventive strategies aimed at promoting physical and mental health by acting on health determinants that are upstream of risk and protective factors and concern environmental, economic, social, behavioral, and cultural aspects of a given population [14]. Primordial preventive interventions are implemented through health and social policies that cover large parts of the general population and could be designed by public health policy makers in collaboration with mental health experts who can provide specific and targeted skills to act effectively on remote determinants of psychiatric disorders. In our research, we have not found scientific publications that have studied the impact of primordial prevention policies on the development of primary psychosis. However, policies of this kind are generally desirable for the prevention of all major and most disabling mental disorders. 

In the second case, we found interventions that target more specific modifiable risk and protective factors in specific subgroups of the population, and they are usually referred as primary preventive inventions [14]. They are also implemented on relatively large portions of the general population and aim to reduce risk factors and enforce preventive ones through public health and health promotion interventions [14]. Unlike primordial prevention policies, they act more specifically on certain factors that are plausibly linked to the pathogenesis of a disorder [14]. As shown above, many studies have validated the role of certain risk factors in aiding the onset of the disorder, but little is known about the effectiveness of policies to reduce them. Few studies evaluated the effectiveness of prenatal and early infancy preventive programs on specific risk factors [88,89]. In particular, nursing home visits to expectant mothers and their families in difficult social circumstances [88] and educational interventions performed at school and at home to teach useful tools to support low-income families and their preschool children [89] were able to reduce child abuse, neglect, and difficult socioeconomic situations through a more profitable use of the welfare system [88,89,90]. Regarding protective factors, by their nature more difficult to investigate and validate from a methodological point of view, the available scientific evidence is much less as compared to the one on risk factors. However, health promotion policies aimed at implementing protective factors of resilience and resistance to the development of mental disorders appear to have had positive effects. An example is that of supportive interventions for mothers with primary psychosis [90]. Such intervention would have been able to reduce the incidence of primary psychosis in the offspring compared to families with equal family vulnerability who had not benefited from this specific health promotion intervention. 

Regarding secondary prevention, which focuses on delaying the onset of the disorder and treating it early, the scientific evidence accumulated to date mainly concerns the study of risk and protective factors with the aim of identifying populations at risk of developing primary psychosis. The rationale for such strategies is to carry out screening programs targeted at restricted segments of the general population where the risk of developing full-blown psychosis within two years is quite high (20–30%). Follow-up on risk subjects enables, on the one hand, to perform policies to reduce risk factors (for example, discouraging the use of cannabinoids) and to promote protective factors (for example, by carrying out targeted psychotherapies) within targeted subpopulations and on the other hand to early detect the onset of the disorder by reducing the DUP. As regards the identification of risk subjects, targeted screening tools have been developed which consider both risk factors and markers, and both modifiable and not modifiable factors. In this case, in fact, the aim is the stratification of the risk in order to make more useful, efficient and less expensive the identification and follow-up of risk subjects. This enables to include risk markers, not necessarily related to the etiology of the disorder, and protective and risk factors not modifiable because, in this case, there are no interventions to act on these factors. It has been demonstrated that screening policies on the general population are not cost/benefit effective. On the contrary, targeted screening gives good results on subpopulation at risk of developing primary psychosis. However, the proportion of primary psychosis with an unexpected onset remains very high and only 5% of total onset of primary psychosis is detected with specific screening programs [90]. For this reason, it is necessary, on the one hand, to refine screening methods and, on the other hand, to maintain active early intervention strategies for early treatment of the onset with effective and evidence-based therapies as soon as possible. To do this, a model is needed that integrates primary care and specialized psychiatric care such as that proposed by Hafner and collaborators and described in the result section [108]. 

Finally, some studies have shown how to act on the risk and protective factors in clinical high-risk groups brings good results in terms of reduction and delay in incidence and better prognosis after the onset. However, again, numerous other studies on large samples are needed. 

The main limitation of the present work is the absence of critical appraisal of the selected papers, as no quality tools were employed in the selection process. This may have biased the selection of scientific work included.

## 5. Conclusions

In conclusion, we can affirm that the study of risk and preventive factors has a central role in improving the early detection of the onset of primary psychosis. It is also crucial for the study of the etiopathogenesis of the disorder and for the implementation of health prevention and health promotion policies that reduce incidence, prevalence and consequently the burden of disability and suffering related to primary psychosis. The study and the knowledge of risk and preventive factors allow the implementation of primordial and primary preventive interventions aimed at reducing risk factors and encouraging protective factors. Preventive interventions, in terms of secondary prevention, allow early detection of the onset of primary psychosis. Overall, prevention and early detection of the onset have positive outcomes including a reduction of the incidence rate of primary psychosis, of the DOI, consequently to the delayed incidence, and of the DUP as a result of prompt interventions at the onset of the disorder. This leads to a prognosis improvement and to a reduction of the burden of disease in terms of disability and suffering for patients and caregivers.

## Figures and Tables

**Table 1:** Gene loci associated with a higher risk of developing primary psychosis.

	Gene Loci	Corresponding Transcript/Protein and Localization	Principal Known Functions			
			*General Description of the Function(s)*	*Central Nervous System (CNS) Development*	*Neuronal Morphology Regulation*	*Signal Transmission*
NEUROTRANSMITTER RECEPTORS	DRD2 (Dopamine Receptor D2)	G protein-coupled membrane receptor	Dopamine signaling			Dopaminergic system
	DRD1, 3, 4 (Dopamine Receptors D1, D3, D4)	G protein-coupled membrane receptor	Dopamine signaling			Dopaminergic system
	HTR2A (5-Hydroxytryptamine -5-HT- Receptor 2A) [44]	G protein-coupled membrane receptor	Regulation of dopamine and glutamate release in the mesolimbic and mesocortical systems			Serotoninergic system Dopaminergic system Glutamatergic system
	GRM3 (Glutamate Metabotropic Receptor 3)	G protein-coupled membrane receptor	Glutamate signaling			Glutamatergic system
	GRIN2B (Glutamate Ionotropic Receptor N-methyl-D-aspartate—NMDA—Type Subunit 2B)	Subunit of the NMDA membrane receptor—ion channel (NMDAR)	Neurodevelopment, circuit formation, synaptic plasticity, and cellular migration and differentiation	Neurogenesis Differentiation of neural progenitors		Glutamatergic system
	GABRB2 (Gamma-Aminobutyric Acid -GABA- Type A Receptor Subunit Beta2)	Subunit of the GABA-A membrane receptor—ion channel	Balance in excitatory/inhibitory signaling in the CNS			GABAergic system
NEUROTRASMITTER METABOLISM AND SIGNAL REGULATION	PDE4B (Phosphodiesterase 4B)	Intracellular enzyme	Cyclic AMP (cAMP)-specific phosphodiesterase (PDE), family IV. It modulates cAMP second messenger concentration in the transduction of the signal process			Signal transmission in different systems
	COMT (Catechol-O-Methyl-transferase)	Intracellular enzyme	Dopamine and noradrenaline metabolism			Dopaminergic system Noradrenergic system
	RGS-4 (Regulator of G Protein Signaling 4) [45]	Intracellular enzyme activator	Activate GTPase activity of G alpha subunits of heterotrimeric G proteins, thus deactivating them	Differentiation of neural progenitors—Axonogenesis in embryogenesis		Glutamatergic system (signal transduction)
	SLC18A2 (Solute Carrier Family 18 Member A2)	ATP-dependent transmembrane transporter	ATP-dependent transporter of monoamines			Dopaminergic system Serotoninergic system Noradrenergic system
	DAOA (D-Amino Acid Oxidase Activator) and DAOA-AS1 (Antisense RNA 1)	Intracellular enzyme activator and corresponding long non-coding RNA	Metabolism of glial D-serine that regulates neuronal NDMAR signaling, mitochondrial function, dendritic arborization		Dendritic regulation	Glia-neuron communication Glutamatergic system
	DTNBP1 (Dystrobrevin Binding Protein 1) and CMYA5 (Cardiomyopathy Associated 5)	Intracellular proteins that participate in the formation of the lysosome-related organelles complex 1 (BLOC-1)	Synaptic vesicle trafficking and neurotransmitter release, in particular regulation of cell surface exposure of DRD2 and glutamatergic release. Actin cytoskeleton reorganization and neurite outgrowth.		Neurite regulation	Dopaminergic system Glutamatergic system Synaptic regulation
CELL-CELL CONTACT AND NEURODEVELOPMENT	DISC1 (Disrupted In Schizophrenia 1)	Scaffold intracellular protein	Involved in neurogenesis (neural progenitor proliferation, neuron positioning), dendritic development, and synapse formation	Neurogenesis	Dendritic regulation	Synaptic regulation
	NRXN1 (Neurexin 1)	Membrane receptor	Binds neuroligins to form Ca(2+)-dependent neurexin/neuroligin complexes at synapses in the CNS			Synaptic regulation
	ERBB4 (Erb-B2 Receptor Tyrosine Kinase 4)	Membrane receptor tyrosine kinases, epidermal growth factor subfamily.	Binds neuroregulins NMDAR pathway	Regulation of CNS development		Glutamatergic system
	NRG1 (Neuregulin 1)	Membrane glycoprotein	Mediates cell–cell signaling. Binds to ERBB receptors NMDAR pathway	Neurogenesis Gliogenesis Differentiation of neural progenitors		Glutamatergic system
	AKT1 (AKT-8 retrovirus Serine/Threonine Kinase 1) [46]	Intracellular Serine/Threonine kinase	Known oncogene involved PI3K and mTOR signaling pathways Placentar and CNS development and functioning	Neurogenesis	Dendritic regulation	Synaptic regulation
	RELN (Reelin)	Large extracellular matrix serine protease	Enzymatic activity modulates cell adhesion	CNS development (cellular migration)	Neuronal morphology regulation (microtubule functioning)	
IMMUNE SYSTEM	IL1B (Interleukin 1 Beta)	Cytokine (extracellular messenger)	Potent proinflammatory cytokine, involved in neuroinflammation during neurodevelopment and adult life	Altered by neuroinflammation		

The genes presented are among the most studied and reported in different meta-analyses or systematic reviews [17,18,22,42,43]. To facilitate consultation of the table, we have divided these genes in relation to their structure, localization, and main function (first column). The last three columns summarize functions synoptically. The nomenclature, the description of the products of the genes and their function is that proposed by GeneCards^®^ [47]. Specific references for the function of single genes are reported in the table.

**Table 2:** Risk and protective factors of primary psychosis.

Factors Associated with the Development of Primary Psychosis		Prenatal	Perinatal	Childhood	Adolescence	Strength of Evidence of the Association	References	Modifiable Factor	Level(s) of Preventive Intervention
**RISK**	Family history of primary psychosis	●				+++	[13,16,17,19,20,21]	No	-
	Paternal characteristics (e.g., age <20 or >45, low socio-economic status)	●				+++	[13,15,23,24]	No	-
	Maternal characteristics (e.g., age <20 or >35, any severe psychiatric condition, pre-pregnancy obesity)	●				+++	[15,23]	Only obesity	Primary
	Diabetes and hypertension during pregnancy		●			++	[15]	Yes	Primary
	Maternal infections during pregnancy		●			+++	[13,23,48]	Yes	Primary
	Suboptimal number of antenatal care visits		●			++	[23]	Yes	Primary
	Maternal stress during pregnancy		●			+++	[13,23]	Yes	Primordial ^a^
	Maternal nutritional deficits during pregnancy		●			+++	[13,23]	Yes	Primary
	Obstetric complications (e.g., maternal or fetal hypoxia during delivery, preterm ruptured membranes)		●			+++	[15,23]	No	-
	Neonatal factors (e.g., premature birth, incubator or resuscitation)		●			+++	[13,15,23]	No	-
	Minor physical anomalies		●			++++	[15,16,24,28]	No	-
	Genetic factors (cf. Table 2)		●	●	●	+++	[17,18,22,42,43]	No	-
	Socio-demographic (e.g., male gender, ethnic minority, birth in winter, disadvantaged groups)	●	●	●	●	++++	[13,15,20,23,24]	Only social part	Primordial ^b^
	Childhood adversities and traumas (e.g., physical, psychological, and sexual abuse, bullying)			●		+++	[13,15,24,51,52,53,55]	Yes	Primordial ^b^
	Difficult parental relationship (e.g., parental communication deviance, separation or death, neglect)			●		+++	[15,52,53,55]	Only some	Primordial ^c^
	Childhood infections (mostly CNS infections)			●		+++	[15,24,28,31,58]	Yes	Primary
	Traumatic brain injury			●	●	++	[13,15]	Yes	Primary
	Exposure to traffic			●	●	++	[15]	Yes	Primordial
	Exposure to benzene			●	●	++	[15]	Yes	Primary
	Tobacco use				●	++	[13,15,24,62]	Yes	Primary/Secondary
	Cannabis use				●	+++	[13,20,24,56,59,60]	Yes	Primary/Secondary
	Psychostimulants use				●	++	[13,20]	Yes	Primary/Secondary
	Autoimmune diseases (e.g., celiac disease, pernicious anemia)			●	●	++	[29,30]	No	-
	Psychological characteristics (e.g., trait anhedonia; premorbid low IQ)			●	●	++++	[15,24]	No	-
	Other psychiatric disorders (e.g., obsessive compulsive disorder, schizotypal personality)				●	++	[32,33,34,35,36,37]	Yes	Primary
	Urbanicity *			●	●	+++	[13,15,20,24,26]	No	-
	Ethnicity (migration and specific ethnic minorities) *			●	●	++++	[15,24]	No	-
**PROTECTIVE**	Prenatal dietary supplementation (vitamin D, iron, folates, phosphatidyl-choline)		●			++	[80,81]	Yes	Primary ^d^
	Socio-demographic (e.g., elevated socio-economic status and an economic well-being)	●	●	●	●	+++	[15]	Yes	Primordial
	Physical characteristics (e.g.,: olfactory identification abilities)			●	●	++	[15,24]	No	-
	Psychological characteristics (e.g., extraversion, openness; high premorbid IQ; resilience)			●	●	++	[16,24,28]	No	-
	Positive family environment (e.g., good parental system, healthy relationships with relatives)			●	●	++	[16,83,84]	Yes	Primordial
	Autoimmune diseases (e.g., ankylosing spondylitis)				●	+	[29,30,79]	No	-
	Physical activity				●	+	[87]	Yes	Primordial/Primary

The number of plus signs denotes the strength of evidence for the association. This number was chosen qualitatively according to the following logic: ++++ indicates very high or high evidence levels reported in meta-analytic umbrella reviews included in this review; +++ indicates consistent evidence reported in multiple large-scale studies or a meta-analysis included in this review; ++ indicates evidence reported in a single meta-analysis included in this review; + indicates evidence reported in a single study, multiple small/low-quality studies, or few studies with conflicting reports included in this review. The strength of the association (e.g., odd ratios, relative risks) reported in the studies mentioned was not reported in the present table. ● indicates the period of life (i.e., prenatal, perinatal, childhood, adolescence) during which risk or protective factors contribute to the development of primary psychosis. NA: not available as we did not find any study that directly analyzed the effect of reducing a risk factor or promoting a protective factor on the development of primary psychosis. * risk marker; ^a^ effective preventive strategies; ^b^ effective preventive strategies [88,89]; ^c^ effective preventive strategies [90]; ^d^ effective preventive strategies [80,81].
